# Hollow Alginate-Poly-L-Lysine-Alginate Microspheres Promoted an Epithelial-Mesenchymal Transition in Human Colon Adenocarcinoma Cells

**DOI:** 10.15171/apb.2020.019

**Published:** 2019-12-11

**Authors:** Shirin Saberianpour, Arezoo Rezaie Nezhad Zamani, Abbas Karimi, Mahdi Ahmadi, Neda Khatami, Ayda Pouyafar, Reza Rahbarghazi, Mohammad Nouri

**Affiliations:** ^1^Department of Molecular Medicine, Faculty of Advanced Medical Sciences, Tabriz University of Medical Sciences, Tabriz, Iran.; ^2^Stem Cell Research Center, Tabriz University of Medical Sciences, Tabriz, Iran.; ^3^Chemical Engineering Faculty, Sahand University of Technology, Tabriz, Iran.; ^4^Department of Applied Cell Sciences, Faculty of Advanced Medical Sciences, Tabriz University of Medical Sciences, Tabriz, Iran.

**Keywords:** Alginate, Epithelial-mesenchymal transition, Human colon adenocarcinoma cell line HT29, Poly-L-lysine, Microspheres

## Abstract

***Purpose:*** Today, there is an urgent need to develop a three-dimentional culture systems mimicking native *in vivo* condition in order to screen potency of drugs and possibly any genetic alterations in tumor cells. Due to the existence of limitations in animal models, the development of three dimensional systems is highly recommended. To this end, we encapsulated human colon adenocarcinoma cell line HT29 with alginate-poly-L-lysine (Alg-PLL) microspheres and the rate of epithelial-mesenchymal transition was monitored.

***Methods:*** Cells were randomly divided into three groups; control, alginate and Alg-PLL. To encapsulate cells, we mixed HT-29 cells (1 × 10^6^ ) with 1 mL of 0.05% PLL and 1% Alg mixture and electrosprayed into CaCl2 solution by using a high-voltage power. Cells from all groups were maintained at 37˚C in a humidified atmosphere containing 5% CO_2_ for 7 days. Cell viability was assessed by MTT assay. To monitor the stemness feature, we measured the transcription of genes such as *Snail, Zeb*, and *Vimentin* by using real-time PCR analysis.

***Results:*** Addition of PLL to Alg in a hallowed state increased the cell survival rate compared to the control and Alg groups (*P*<0.05). Cells inside Alg-PLL tended to form microcellular aggregates while in Alg microspheres an even distribution of HT-29 cells was found. Real-time PCR analysis showed the up-regulation of *Snail, Zeb*, and *Vimentin* in Alg-PLL microspheres compared to the other groups, showing the acquisition of stemness feature (*P*<0.05).

***Conclusion:*** This study showed that hallow Alg-PLL microspheres increased the epithelialmesenchymal transition rate after 7 days in *in vitro* condition. Such approaches could be touted as appropriate *in vitro* models for drug screening.

## Introduction

Up to the present time, many attempts have been made to investigate the type and entity of extracellular matrix (ECM) on cancer cells dynamics by using two (2D) and three dimensional (3D) substrates.^1^ There are numerous peptides and proteins playing as modulators of cancer cells behavior and even could act as growth factors during the development and progression of various tumor types.^2^ It was shown that distinct ECM could transmit survival signals between cancer cells.^3^ In this regard, it was elucidated that these proteins had potential to dictate polarity and phenotype acquisition to the neoplastic cells by triggering a phenomenon termed as epithelial-to-mesenchymal transmission (EMT), causing tumor resistance to the anticancer agents and therapies.^4^ This phenotypic switching increases the number of cancer progenitor cells so-called cancer stem cells (CSCs).^5^ EMT is regulated by a number of functional proteins inside cancer cells that act as polyoleptic agents such as ZEB, Snail, and Vimentin, etc. The activity of these genes performs an important milieu for the expression of markers associated with mesenchymal cells phenotype.^6^

In recent years, the microencapsulation technique has been used extensively to create a 3D microenvironment in the field of cancer biology and tissue engineering.^7^ Basically, alginate (Alg), a natural polysaccharide, and to less extent other substrates such as poly-l-lysine (PLL), are extensively used for the preparation of microspheres.^8^ Alg is potential to cross-link with different substrates in the presence of calcium and barium ions and functions as the basic scaffold to create a membrane shell protecting cells from external injuries.^9^ Based on the scientific literature, the main reason for the use of PLL is to increase resistance around the center of the microspheres.^10^ This resistance is correlated with the reduction of swelling rate and membrane shell pore size.^11^ The main goal of this study was to investigate the occurrence of EMT in the human colorectal adenocarcinoma cell line (HT-29) after being encapsulated by the mixture of Alg and PLL. The cells were maintained inside the microspheres containing Alg alone and the mixture of Alg and PLL. The advantage of this method can be related to create a 3D environment for human cancer cells and study the possibility of EMT in HT-29 cells inside the Alg-PLL microspheres.

## Materials and Methods

### Cell culture

Human colorectal adenocarcinoma cell line; HT-29, was purchased from Iranian Cell Bank (Pasteur, Iran). Cells were grown in high glucose-content Dulbecco’s Modified Eagle Medium (DMEM/HG; Cat no: 31600-083; Gibco; Carlsbad; CA; USA) with 10% v/v fetal bovine serum (FBS; Cat no: 10270; Gibco; Carlsbad, USA) and 1% v/v penicillin-streptomycin (Pen-Strep; Cat no: 10378016; USA) in culture flasks. Cells were sub-cultured at 70%-80% confluence by using 0.25% Trypsin and 1 mM EDTA solutions (Cat No: 25200-056; Gibco; USA) solution. Cells were subjected to the current experiments at passages three to six.

### Microencapsulation of HT-29 cells using Algand the PLL mixture

To explore the potency of ECM type on the stemness and multipotentiality of HT-29 cells, we encapsulated cells with the combination of Alg (1% w/v; Cat no: A2033; Sigma-Aldrich; Germany) and PLL (0.05% w/v; Cat no: P8920; Sigma-Aldrich; Germany) by using electrospray device (FnM, Hu35p oc, Nigh voltage power supply). To this end, we classified the cells into three different groups as follows; Control: HT-29 cells were expanded on the conventional plastic surface; Alg group: HT-29 cells were encapsulated by 1% Alg solution and Alg + PLL group: cells were enclosed by the mixture of Alg and PLL. To encapsulate cells, 1 × 10^6^ HT-29 cells were mixed with 1 ml of 1% Alg and transferred into a sterile syringe with 26G-gauge needle. Thereafter, the cells suspension was poured into a syringe pump connected to an electrospray device, adjusted to a voltage of 8 kV. Dropping the cell mixture into a 1% CaCl_2_ (Cat no: 1.02382.1000; Darmstadt; Germany) solution contributed to the formation of microcapsules. To exclude CaCl_2_, we washed the microcapsules by CF-KRH solution (0.48% HEPES, 0.79% NaCl, 0.35% KCl, 0.1% Glucose). In Alg + PLL group, we added PLL to the Alg backbone. To remove the Alg from Alg + PLL microspheres, we treated these microcapsules with 0.02% EDTA (Cat no: 60-00-4; Sigma-Aldrich, Germany) for 5 min at 37°C by using CF-KRH solution. Cells from all groups were incubated at 37°C in a humidified atmosphere containing 5% CO_2_ for 7 days. Seven days after encapsulation, the cells were released from microcapsules by incubating cells with 0.01% sodium citrate (Cat no: 6132-04-3; Merck, Darmstadt; Germany). Electrospraying procedure was carried out under sterile condition.

### Cell Survival detection by MTT assay

The survival of HT-29 cells from each group was evaluated by MTT assay. For this propose, 3000 cells were mixed with 100 μL of MTT solution (Cat no: 298-93-1; Sigma-Aldrich; Germany) and poured into each well of 96-well plates and kept at 37°C conditions for 4 hours. Then, 50 μL of dimethyl sulfide (DMSO; Merck, Darmstadt, Germany) solution were added into each well and plates gently agitated for 15 min. Finally, the optical density of each well was read by using an ELISA reader (Model: Ex808, BioTek, USA) at a wavelength of 570 nm.

### Real-time PCR

HT-29 cells were collected from each group and total RNA content isolated by RNA extraction kit (Cat no: FABRK001; Yekta Tajhiz Azma, Iran). The purity of isolated RNAs was evaluated using a Thermo Scientific Nanodrop^TM^ 1000 system. Subsequently, RNAs were converted cDNA by using cDNA synthetase (Bioneer). In this study, we monitored the transcription of three genes Snail, Vimentin, and Zeb by appropriate primers designated by Oligo 7 software (version 7.60) (Table 1). The level of gene expression was measured by SYBR Green and Mic Real-Time PCRSystem. The expression of each gene was normalized to the housekeeping gene GAPDH.

**Table 1:** Primer list

**Gene**	**Sequences (5' -> 3')**	**Accession No.**	**Temperature (°C)**	**Length (bp)**
VIM	F:CAGATGCGGTGAAATGGAAGAGAA	NM_003380.5	61	174
	R:TAGGTGGCAATCTCAATGTCAA			
ZEB1	F:CTGGAGAAAAGCCCTATCAATGT	NM_001323671.2	59.7	244
	R: CTGTCTTCATCCTCTTCCCTTGT			
SNAI1	F: TAGCGAGTGGTTCTTCTGCG	NM_005985.4	60	164
	R: AGGGCTGCTGGAAGGTAAAC			
GAPDH	F:CCTGCACCACCAACTGCTTA	NM_001289745.3	60	95
	R: AGTGATGGCATGGACTGTGG			

## Results and Discussion

The transition to invasive and metastatic subpopulations consists of changes in tumor cells function.^12^ To attain these characteristics, morphogenetic transformations, known as EMT, is in an active state that maintains the balance between epithelial phenotype and stemness feature.^13^ The application of 3D culture system could mimic to some extents the *in vivo* conditions.^14^

### Cell morphology and appearance

**Figure 1:**
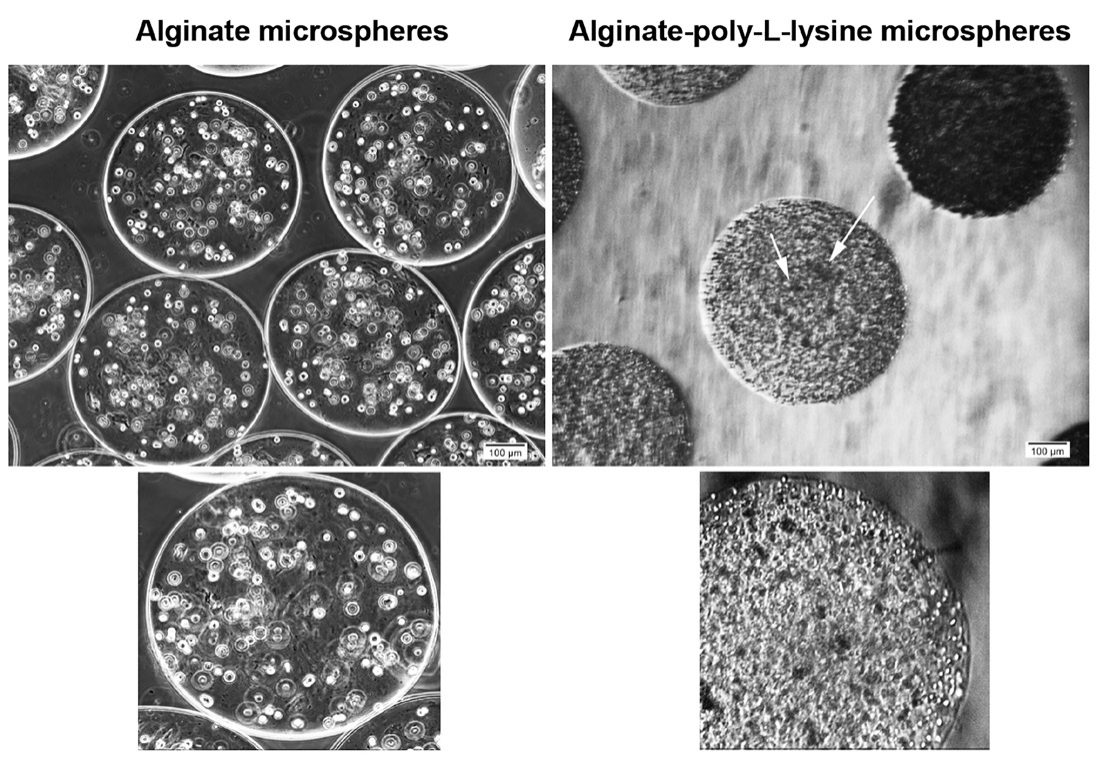
Bright-field microscopic imaging of HT29 cells after 7-day encapsulation with alginate and alginate-poly-l-lysine. As shown, cells are evenly distributed inside the alginate microspheres while these cells tend to form cellular microaggregates in the matrix of alginate-poly-l-lysine (arrow = cellular microaggregates).

According to our data, HT-29 cells acquired a round shape morphology and were evenly distributed inside Alg microspheres (Figure 1). To elucidate the role of PLL on cell dynamic, we removed Alg from Alg + PLL microspheres core by using EDTA solution (a hallowed structure) while the membrane shell remained and consisted of PLL and Alg. In hallowed microspheres, HT-29 cell tended to form microaggregates after 7 days (Figure 1). The data showed that encapsulation of HT-29 cells with microspheres consisted of Alg distributed cells evenly over 7 days while Alg removal and enclose of cells with Alg + PLL membrane contributed to the formation of cellular microaggregates.

### PLL + Alg encapsulation increased HT-29 cells survival rate

**Figure 2:**
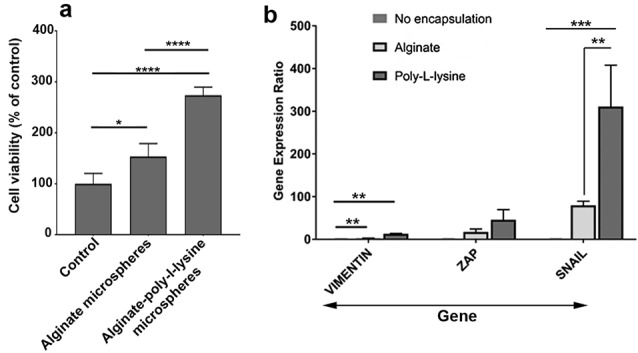
Measuring cell viability after 7-day encapsulation with alginate and alginate-ploy-l-lysine (**a**). Compared to the control, the viability of cells was increased inside the alginate and alginate-poly-l-lysine microspheres. Based on the results, the addition of poly-l-lysine to alginate increased cell survival (n = 3). Real-time PCR assay showed the expression of all genes *Vimentin*, *Zeb* and *Snail* compared to the control and alginate group (n = 3). One-way ANOVA and Tukey post hoc analysis. * *P* < 0.05; ** *P* < 0.01; *** *P* < 0.001 and **** *P* < 0.0001.

MTT assay showed an increased HT-29 cells survival rate after being encapsulated with Alg alone or Alg+ PLL microspheres compared to the control HT-29 cells expanded on the plastic surface (Figure 2a). Compared to the control, an approximately 3-fold increase was recorded in the survival rate for HT-29 cells inside Alg + PLL microspheres (*P* < 0.0001) while lower survival rate was evident in Alg group (*P* < 0.05). It seems that the addition of PLL to Alg backbone had the potential to increase the HT-29 cell survival rate (*P* < 0.0001; Figure 2a). These data showed that the encapsulation of human adenocarcinoma HT-29 cells with Alg and especially with the combination of Alg + PLL promoted cell survival rate. Based on the previously published data, it seems that the addition of PLL to Alg and development of hallow environment could reduce membrane shell pore size and decrease Alg swelling rate while increasing cell-to-cell interaction in 3D condition.^15^ Due to the existence of negative charges in Alg microspheres, the juxtacrine interaction of tumor cells would decrease.^16^

### Microencapsulation increased EMT rate

To monitor the potency of encapsulation on EMT and stemness acquisition, we also performed real-time PCR analysis.^17^ Data showed that the expression of three genes Snail, Vimentin and Zeb were increased 7 days after encapsulation of HT-29 cells with Alg and PLL compared to the control cells expanded on the plastic surface (*P* < 0.05; Figure 2b). Of note, the use of Alg microspheres loaded with PLL could significantly up-regulate all genes (Figure 2b). These genes participate in the EMT process. Consistent with these results, one could hypothesize that the development of a 3D niche consisted of distinct peptides such as PLL could induce the stemness feature and multipotentiality in cancer cells. Consistent with our result, it was demonstrated that soluble PLL could induce necrosis and apoptosis in tumor cells by recruiting monocytes and inflammatory cells. Various facts highlighted the emergence of CSCs in response to an insulting condition such as immune-related activity.^18^

Physical contact is essential for cells bioactivities such as proliferation, cell migration, and differentiation.^19^ These events were promptly facilitated by neighboring scaffolds.^20^ Even, the type of ECM participates in the regulation of cell expansion, migration, and survival. The entity of ECM could activate mechanical receptors on the surface of cancer cells which further activates downstream signaling pathways.^21^ In this regard, due to the existence of cationic amino acid, profound cellular connectivity and cellular aggregation are provided by PLL.^22^ Cellular adhesion was also stimulated via the strong electrostatic bond between positively charged PLL and the negative charge of cell surface.^23^ Qi et al previously showed that the combination of PLL with graphene oxide not only could increase the proliferation of mesenchymal stem cells but also dictated trans-differentiation into osteoblast-like cells.^24^ The stimulatory effect of PLL could be related in part to its physicochemical activity. It is postulated that the existence of positive charges on the PLL generates an electrostatic interaction with negative elements of the cell membrane.^25^ In support of this statement, *in vitro* culture of hematopoietic stems cells on PLL substrate increased the number of cells entering S phase.^25^

## Conclusion

Based on the entity and composition of ECM, the phenotype of tumor cells could be changed in the 3D milieu. Therefore, the use of Alg-based components could be advised to the promotion of specific and distinct cell type in in vitro and in vivo milieu.^26^ The current experiment highlighted the stimulatory effect of PLL on EMT and phenotype shifting.

## Ethical Issues

This article does not contain any studies with human participants or animals performed by any of the authors.

## Conflict of Interest

The authors declare that they have no conflict of interest.

## Acknowledgments

We would like to thank the personnel of the Stem Cell Research Center for guidance and help. This work was financially supported by a grant from Tabriz University of Medical Sciences.
